# Stratified care for patients with sciatica and suspected sciatica in primary care: the scopic trial protocol (scopic - sciatica outcomes in primary care)

**DOI:** 10.1186/1745-6215-16-S2-P156

**Published:** 2015-11-16

**Authors:** Nadine Foster, Kika Konstantinou, Ruth Beardmore, Kate Dunn, Martyn Lewis, Bernadette Bartlam, Majid Artus, Elaine Hay

**Affiliations:** 1 Keele University, Keele, Staffordshire, UK

## Background

Usual care for sciatica is ‘stepped’ and starts with advice, analgesia and ‘wait and see’, where patients are only referred to other services if symptoms fail to improve. We have shown the superiority of stratified care (subgrouping for targeted treatment) over usual care for non-specific back pain. We now aim to test a new model of stratified care for patients with sciatica.

## Methods

In a pragmatic RCT with 470 primary care sciatica patients, participants will be randomised to stratified care or usual care. Stratification will be based on a combination of 4 clinical indicators (interference with work-ability, pain below the knee, intense leg pain and sensory deficits) that predict referral to spinal specialists and 8 prognostic indicators of persistent disability (STarT Back tool). Patients will be allocated to 1 of 3 subgroups: those at low risk will receive advice and support to self-manage; those at medium risk will receive a course of physiotherapist-led treatment, and those at high risk will be ‘fast-tracked’ to spinal specialists. Primary outcome is time to symptom resolution collected using regular SMS text messages. Linked qualitative interviews will be conducted with patients (and clinicians) in the ‘fast-track’ pathway to explore it's acceptability.

## Results

Primary analysis will compare time to resolution between stratified care and usual, non-stratified care, on an intention-to-treat basis, and cost-effectiveness will also be investigated. Kaplan-Meier survival analysis and Cox regression analysis will be used. Qualitative interviews will be analysed thematically.

## Conclusions

This is the first RCT to test a model of stratified primary care for patients with sciatica and suspected sciatica.

